# From Detection to Motion-Based Classification: A Two-Stage Approach for *T. cruzi* Identification in Video Sequences

**DOI:** 10.3390/jimaging11090315

**Published:** 2025-09-14

**Authors:** Kenza Chenni, Carlos Brito-Loeza, Cefa Karabağ, Lavdie Rada

**Affiliations:** 1 Department of Electronics, Faculty of Technology, University Ferhat Abbas Sétif 1, Sétif 19000, Algeria; kenza.chenni@univ-setif.dz; 2 Computational Learning and Imaging Research, Facultad de Matemáticas, Universidad Autónoma de Yucatán, Mérida 13615, Mexico; carlos.brito@correo.uady.mx; 3 Department of Computer Science, School of Science & Technology, City St George’s, University of London, London EC1V 0HB, UK; cefa.karabag@citystgeorges.ac.uk; 4 Faculty of Engineering and Natural Sciences, Bahçeşehir University, İstanbul 34349, Türkiye

**Keywords:** Chagas disease, *T. cruzi*, motion detection, deep learning, YOLO, automated diagnosis, microscopy

## Abstract

Chagas disease, caused by *Trypanosoma cruzi* (*T. cruzi*), remains a significant public health challenge in Latin America. Traditional diagnostic methods relying on manual microscopy suffer from low sensitivity, subjective interpretation, and poor performance in suboptimal conditions. This study presents a novel computer vision framework integrating motion analysis with deep learning for automated *T. cruzi* detection in microscopic videos. Our motion-based detection pipeline leverages parasite motility as a key discriminative feature, employing frame differencing, morphological processing, and DBSCAN clustering across 23 microscopic videos. This approach effectively addresses limitations of static image analysis in challenging conditions including noisy backgrounds, uneven illumination, and low contrast. From motion-identified regions, 64×64 patches were extracted for classification. MobileNetV2 achieved superior performance with 99.63% accuracy, 100% precision, 99.12% recall, and an AUC-ROC of 1.0. Additionally, YOLOv5 and YOLOv8 models (Nano, Small, Medium variants) were trained on 43 annotated videos, with YOLOv5-Nano and YOLOv8-Nano demonstrating excellent detection capability on unseen test data. This dual-stage framework offers a practical, computationally efficient solution for automated Chagas diagnosis, particularly valuable for resource-constrained laboratories with poor imaging quality.

## 1. Introduction

Chagas disease, a life-threatening disease caused by the protozoan parasite *T. cruzi*, affects approximately 6 to 7 million people worldwide, with a much higher number at risk of infection [1]. Although traditionally endemic to Latin America, global migration patterns have amplified the disease’s reach, spreading it to non-endemic areas [2]. As a neglected tropical disease, Chagas disease continues to pose a significant public health challenge, particularly in regions with limited access to early diagnosis and treatment.

The primary mode of transmission occurs through the faeces of blood-feeding triatomine insects, commonly known as “kissing bugs”. However, the disease can also spread through alternative routes, including blood transfusions, organ transplants, and the consumption of contaminated foods and beverages [2,3]. This multifaceted transmission pattern contributes to the complexity of disease control and prevention strategies.

**Figure 1:**
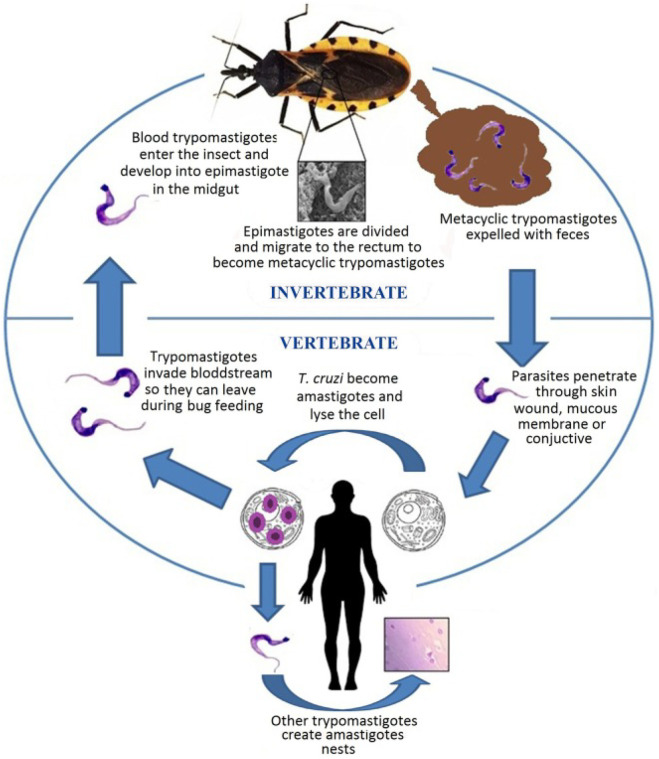
Life cycle of *T. cruzi*. The image is taken from the work of De Fuentes-Vicente et al. [8].

*T. cruzi*, the aetiological agent of Chagas disease, is a flagellated protozoan parasite belonging to the order Kinetoplastida [4]. This microscopic organism exhibits a complex life cycle that alternates between insect vectors and mammalian hosts [5]. In the digestive tract of triatomine insects, *T. cruzi* exists in its epimastigote form, where it undergoes reproduction. Subsequently, it transforms into the infective metacyclic trypomastigote form, which is transmitted to humans and other mammals through the insect’s faeces [6]. Once inside the mammalian host, the parasite invades cells, metamorphoses into its amastigote form, and multiplies intracellularly [7]. Figure 1 provides a comprehensive illustration of the entire life cycle of *T. cruzi*, depicting its development within the triatomine insect vector, the transformation into infectious forms, and the intracellular replication stages in mammalian hosts. This intricate life cycle and the parasite’s ability to evade host immune responses contribute to the complexity of Chagas disease pathogenesis and the challenges associated with its diagnosis and treatment.

The clinical manifestations of Chagas disease are diverse and can be categorized into acute and chronic phases. The acute phase, often asymptomatic or characterised by mild, non-specific symptoms, can last for several weeks or months. If left untreated, the disease progresses to a chronic phase, which can remain asymptomatic for decades in most infected individuals. However, approximately 30–40% of patients develop severe cardiac or gastrointestinal complications, leading to significant morbidity and mortality [9]. The chronic cardiac form of Chagas disease is particularly concerning, as it can result in heart failure, arrhythmias, and sudden death [10]. This progression underscores the importance of early detection and intervention. Early and accurate diagnosis is crucial for the effective treatment and management of Chagas disease. The chances of successful treatment are significantly higher when intervention begins during the acute phase [11].

Traditional diagnostic methods, such as serological tests, blood smear microscopy, and polymerase chain reaction (PCR), are limited by poor accessibility, high costs, and the need for specialized equipment [12]. These limitations hinder early detection, particularly in resource-limited settings where Chagas disease is most prevalent. For this reason, the theme of World Chagas Disease Day 2022 was “finding and reporting every case to defeat Chagas disease” [1]. This highlights the urgent need for innovative, accessible, and accurate diagnostic tools that can overcome these barriers and support timely interventions.

Recent advances in understanding *T. cruzi* biology and Chagas disease pathogenesis have spurred research into novel diagnostic approaches. Bern et al. [13] underscored the need for sensitive and specific tools in their review of current diagnostic practices, while Zingales et al. [14] highlighted the importance of standardised nomenclature for improving diagnosis and treatment strategies. In this context, deep learning-based solutions have emerged as a promising avenue for addressing these challenges.

However, existing automated approaches face significant limitations in real-world clinical settings. Static image analysis methods achieve only modest accuracies: Pereira et al. [15] reported 72–96.4% accuracy with substantial performance drops on unseen test data due to over-fitting, while Morais et al. achieved 89.5% accuracy using traditional machine learning classifiers. These appearance-based methods rely on time-consuming staining protocols and fail under poor lighting conditions common in field microscopy. Moreover, morphological features alone are often indistinguishable from background artifacts in unstained blood samples, creating diagnostic challenges in resource-limited settings where Chagas disease is prevalent.

Current diagnostic workflows create critical barriers to rapid diagnosis. The lengthy staining process delays treatment decisions, particularly during the acute phase when intervention is most effective. Manual microscopy remains subjective and requires specialized expertise unavailable in many endemic regions.

Our motion-based framework addresses these limitations by eliminating staining requirements through motion analysis of live, unstained blood samples, enabling rapid real-time diagnosis. It provides robust performance under variable lighting and imaging conditions and achieves high diagnostic accuracy (99.63%) in our evaluation, highlighting its potential to significantly outperform existing methods. Furthermore, its lightweight design supports deployment on portable devices for point-of-care diagnosis in resource-constrained environments.

In this study, we propose a novel computer vision framework for automated *T. cruzi* parasite detection, emphasizing a motion-based classification approach that directly addresses the limitations of conventional static frame analysis. Recognising the diagnostic challenges posed by complex microscopic environments—including heterogeneous backgrounds and variable lighting—we shift the focus from appearance-based object detection to motion-centric temporal analysis. Our methodology integrates adaptive frame differencing, motion saliency enhancement, and advanced contour-based clustering techniques to isolate parasite-specific movement patterns, thereby enabling robust ROI generation for deep learning-based classification. To validate our approach, we constructed a balanced dataset from 23 microscopic video sequences and evaluated performance using several deep learning architectures, including MobileNetV2, AlexNet, and VGG16. As a comparative baseline, we implemented a YOLO-based parasite detection model trained on 48 manually annotated microscopic video sequences. While YOLO-based appearance models have demonstrated strong performance in general computer vision tasks, they often encounter limitations in microscopic environments, where low contrast and cluttered backgrounds obscure clear object boundaries. In contrast, our motion-focused framework which leverages temporal dynamics and flagellar movement proved highly reliable under the same challenging conditions, underscoring the critical importance of motion-based cues for accurate and consistent identification of *T. cruzi* parasites in complex microscopic environments.

The remainder of this paper is organized as follows: Section 2 reviews related work in parasite detection and motion-based computer vision. Section 3 describes the materials and methods, with detailed attention given to our motion-based classification framework. Section 4 presents the experimental results, including both classification performance of the motion-based framework and detection performance of the YOLO-based approach. Section 5 concludes with a discussion of the implications of this work for scalable, automated diagnosis of Chagas disease.

## 2. Related Work

The limitations of traditional diagnostic methods for Chagas disease have prompted the exploration of more accurate, innovative approaches. The approaches consist of a simple blood smear technique, a cost-effective procedure, and automated analysis methods for efficient and accurate detection. Similar to advancements in other fields, the broader discipline of parasitology has witnessed transformative progress with the integration of machine learning and artificial intelligence. Parallel to the development of new, simple blood smear techniques as cost-effective diagnostic procedures, these technologies have significantly enhanced diagnostic accuracy and enabled the rapid generation of results.

For instance, Soberanis-Mukul et al. [16] presented a machine learning algorithm for *T. cruzi* parasite automatic detection. When evaluated against a dataset of 120 test images, their method yielded a sensitivity of 98% and a specificity of 85%. However, the study acknowledged challenges related to the algorithm’s reliance on image inputs and its susceptibility to variations in image colour due to different colourisation procedures.

Uc-Cetina et al. [17] proposed a detection method using the AdaBoost algorithm to identify Chagas parasites in blood images, achieving a sensitivity of 100% and specificity of 93.25%. Rosado et al. [18] reviewed various image processing and machine learning techniques for the automatic detection and segmentation of malaria parasites in microscopic images of blood smears. While their method focuses on detecting the presence of the parasite, our work extends this by using classification techniques that not only detect but also categorise the images into specific classes, such as infected versus non-infected. This comparison highlights the effectiveness of machine learning in both detection and classification tasks, with detection serving as an important precursor to the classification process in parasitic disease diagnosis.

Pereira et al. [15] proposed a computational strategy for the automated classification of *T. cruzi* parasites in microscopic blood samples. Using a pre-trained MobileNetV2 neural network, they developed a feature extraction system, which was then fed into a specially designed single-cell binary classification layer. This innovative architecture demonstrated remarkable effectiveness, achieving an accuracy rate of up to 96.4% on the validation dataset. However, they observed a significant drop in test accuracy to 72%, which they attributed to over-fitting and the limitations of the small dataset.

Morais et al. [19] presented a classification framework that relies on preprocessing techniques such as segmentation to extract regions of interest, followed by feature extraction using geometric, texture, and colour descriptions. Their study compared machine learning classifiers such as SVM [20], KNN [21], and Random Forest (RF) [22], where the RF model achieved a test accuracy of 89.5% and an AUC of 0.942. They also highlighted challenges such as over-fitting, dataset size, and image diversity.

Motion analysis has emerged as a powerful alternative to appearance-based parasite detection, particularly in dye-free or low-contrast microscopy where the morphology of *T. cruzi* is often indistinguishable from background artifacts. Martins et al. [23] introduced a biologically inspired approach that estimates dense optical flow, converts it into saliency maps, and leverages collateral motion cues. They found their model to be a promising tool in the research and medical diagnosis of Chagas disease.

In parallel, researchers in video surveillance have demonstrated the efficiency of lightweight motion techniques under constrained hardware. Thapa, Sharma, and Ghose [24] proposed a Differencing and Summing Technique (DST), which computes frame wise differences and aggregates multi-frame residuals to suppress background noise. Their method yielded reliable segmentation in both indoor and outdoor scenes, with minimal computational load. Similarly, Husein et al. [25] showed that single threshold frame differencing enables real-time motion detection on embedded systems.

Building on these insights, our framework applies temporal motion analysis through frame differencing to isolate dynamic objects, specifically, motile *T. cruzi* parasites in microscopic video sequences. Unlike conventional static-image detection methods, our motion centric pipeline exploits pixel level intensity changes across consecutive frames to identify active regions. This is followed by morphological operations and contour-based filtering to eliminate noise and localise parasite candidates. The resulting approach is robust to cluttered or poorly illuminated imaging conditions and remains lightweight enough for deployment in resource-limited diagnostic environments.

## 3. Materials and Methods

The primary data source consisted of digital camera video recordings capturing *T. cruzi* parasites. Unlike traditional methods, where *T. cruzi* detection relies on examining microscope images during the acute phase of infection—a process in which specialists manually prepare thin blood smears, stain them, and examine them under a microscope (a labour-intensive procedure taking approximately 20 min per sample)—the new dataset provided by the Universidad Autónoma de Yucatán offers a more efficient approach. This dataset consists of centrifuged blood samples (500 rpm for 2 min), with microscopy analysis performed within 5 min of blood collection, enabling a faster and less laborious examination compared to conventional methods.

### 3.1. Database

A Canon EOS Rebel Tli (Model 500D) digital camera was used for video recording, capturing the dynamic behaviour of and morphological changes in *T. cruzi* in an infected mice blood sample. The camera was connected via a C-mount adapter without optics and had a resolution of 1280×720 pixels, and was operated manually without microscopy image analysis software. The experiment was conducted by the Universidad Autónom de Yucatán, and all techniques for animal handling and sample collection were approved by the Institutional Animal Care and Use Committee of the University (IACUC-03—2021). For the experiments, infected mice were used, with a parasite concentration of 3 million parasites per millilitre. Blood samples were collected using 75 mm long capillary tubes with a 30-microlitre volume, directly at the puncture site or from EDTA tubes after venipuncture. The samples were centrifuged at 500 rpm for 2 min, and microscopy analysis was performed within 5 min of blood collection.

### 3.2. From Video Processing to Motion-Based Classification

**Figure 2:**
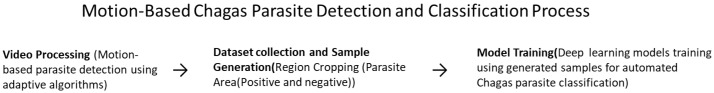
Motion-based Chagas parasite detection and classification framework. The process comprises video preprocessing, candidate region extraction, sample generation, and deep learning model training.

As illustrated in Figure 2, the proposed Chagas parasite detection framework comprises four key stages: video processing, model training, evaluation, and findings.

Finding a technique that can be easily implemented on simple computing devices is crucial for practical deployment in real-world settings, especially in low-resource laboratories or mobile diagnostic systems. In this work, we propose a lightweight, motion-based method that enables the efficient detection of moving objects, specifically, parasitic organisms in microscopic video sequences. The method emphasizes simplicity, robustness, and computational efficiency to ensure wide applicability. To achieve this, we developed an innovative computer vision framework that combines adaptive motion detection with intelligent filtering and morphological analysis to automatically identify parasites in optical microscopy datasets.

**Figure 3:**
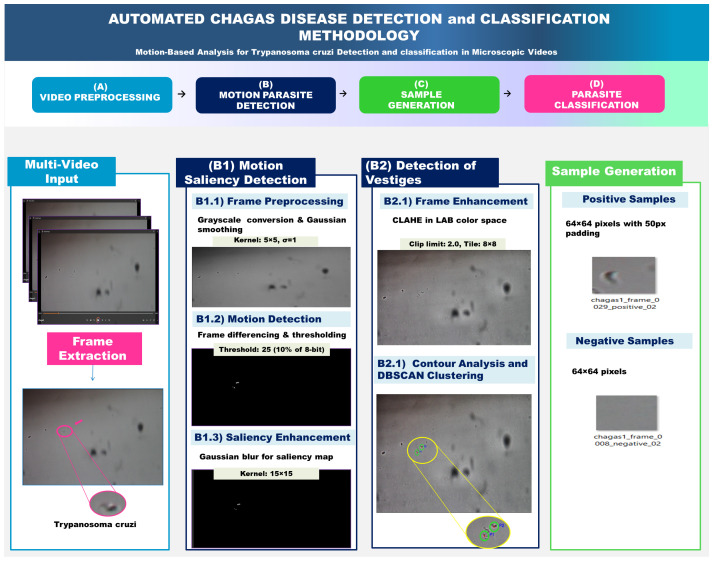
Comprehensive pipeline for automated Trypanosoma cruzi detection and classification. This diagram outlines the four key stages of a motion-based analysis methodology for identifying and classifying Trypanosoma cruzi parasites from microscopic video data. (**A**) Video Preprocessing extracts frames from multi-video input. (**B**) Motion Parasite Detection uses a two-part approach: (**B1**) Motion Saliency Detection identifies moving objects, and (**B2**) Detection of Vestiges refines these detections using frame enhancement and DBSCAN clustering. The detected parasites are then used in (**C**) Sample Generation to create a balanced dataset of positive and negative samples (e.g., 64 × 64 pixel images) for subsequent machine learning. This dataset is then used in (**D**) Parasite Classification to train a model for the automated diagnosis of Chagas disease.

Figure 3 illustrates the complete methodology for parasite detection using adaptive motion detection. The pipeline consists of three main phases: Multi-Video Input Processing, Motion Saliency Detection, and Trace Detection, culminating in automated sample generation for machine learning applications.

#### 3.2.1. Adaptive Motion Detection Algorithm and Preprocessing

##### Frame Differencing Technique

The Frame Differencing Technique is a computationally simple yet highly effective method widely used in computer vision for detecting motion. A video is composed of a sequence of frames, each made up of pixels with RGB values ranging from 0 (black) to 255 (white). By subtracting two consecutive frames pixel-wise, static regions where pixel values remain unchanged appear black, while regions with movement manifest as bright areas due to changes in pixel intensity. This fundamental concept serves as the basis for motion detection in video sequences.

**Figure 4:**
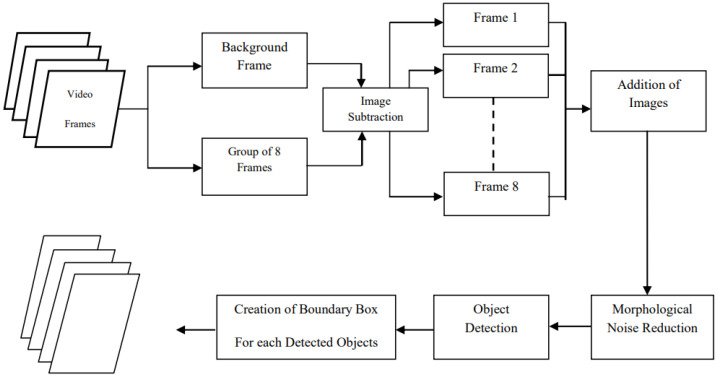
Block diagram demonstrating the sequence of steps in moving object detection using frame differencing [24].

Figure 4 illustrates the steps for moving object detection using frame differencing.

#### 3.2.2. Algorithm Implementation

##### Frame Differencing Algorithm

The Frame Differencing Technique employs temporal analysis to detect parasite motion by comparing consecutive video frames and employment of different image processing techniques as shown in Algorithm 1.
**Algorithm 1** Motion Detection using Frame Differencing**Require:** Video sequence $\left\{I_{1},I_{2},\ldots ,I_{n}\right\}$
**Ensure:** Motion saliency maps $\left\{S_{1},S_{2},\ldots ,S_{n-1}\right\}$
 1: **for** each frame pair $\left(I_{n-1},I_{n}\right)$ **do**
 2:     Convert frames to grayscale
 3:     Apply Gaussian smoothing (5×5 kernel)
 4:     Calculate absolute difference: $\Delta_{n}\left(x,y\right)=\left|I_{n}\left(x,y\right)-I_{n-1}\left(x,y\right)\right|$
 5:     Apply threshold: $M_{n}\left(x,y\right)=\{\begin{array}{cc} 255 & \mathrm{if}\Delta_{n}\left(x,y\right)>T\\ 0 & \mathrm{otherwise} \end{array}$
 6:     Morphological filtering (opening + closing, 3×3 ellipse)
 7:     Generate saliency map: $S\left(x,y\right)=G_{\sigma =15}*M\left(x,y\right)$
 8: **end for**


Table 1 summarizes the key parameters used in our implementation as Algorithm 1 is applied.

##### Frame Enhancement

Contrast-limited adaptive histogram equalization (CLAHE) is applied to enhance parasite visibility before training samples are extracted. The enhancement process modifies the L-channel (lightness) while preserving chromaticity information, as detailed in Algorithm 2.
**Algorithm 2** Adaptive Frame Enhancement**Require:** RGB frame *I*
**Ensure:** Enhanced frame $I_{enhanced}$
   1: Convert *I* from BGR to LAB colour space
   2: Split into *L*, *A*, *B* channels
   3: Apply CLAHE to *L*-channel:
   4:      $L^{\prime }=\mathrm{CLAHE}\left(L,\mathrm{clip}\_\mathrm{limit}=2.0,\mathrm{tile}\_\mathrm{size}=8\times 8\right)$
   5: Merge enhanced $L^{\prime }$ with original *A*, *B* channels
   6: Convert back to BGR colour space
   7: **return** 
$I_{enhanced}$


Table 2 shows the enhancement parameters.

##### Parasite Detection and Localization

The detection pipeline combines contour analysis with DBSCAN clustering to identify and refine parasite locations, as detailed in Algorithm 3. Table 3 outlines the key parameters.
**Algorithm 3** Parasite Detection Pipeline**Require:** Saliency map S(x,y), Enhanced frame *I*
**Ensure:** Refined parasite locations $\left\{C_{1},C_{2},\ldots ,C_{k}\right\}$
 1: Threshold saliency map (threshold = 30)
 2: Apply morphological operations (2×2 kernel)
 3: Find contours using Suzuki-Abe algorithm
 4: Filter contours by area (A>50 pixels)
 5:**for** each valid contour **do**
 6:     Calculate moments $M_{00}$, $M_{10}$, $M_{01}$
 7:     Centroid: $\left(c_{x},c_{y}\right)=\left(M_{10}/M_{00},M_{01}/M_{00}\right)$
 8: **end for**
 9: Apply DBSCAN (ε=30, min_samples = 2) to centroids
 10: Calculate cluster centroids as final detections
 11: **return** refined parasite locations


In general, preprocessing techniques are computationally expensive, and the primary concern when applying them is the required processing time. Table 4 presents a detailed time analysis of the motion preprocessing stage. The reported results include both the total processing time for 23 videos and the average time per video. As shown, the preprocessing step requires only a few seconds per video, demonstrating that while it is time-consuming in general, the per-video cost remains relatively small.

##### Training Data Generation

Our experimental setup utilized 23 microscopic video sequences containing the parasite *T. cruzi*. Each video was processed as described in the previous sections, including frame extraction, motion analysis, parasite detection, and sample generation. Positive training samples are extracted by cropping 64×64 pixel regions around detected parasite locations. Each crop is padded by 50 pixels to ensure complete parasite capture, as defined in Algorithm 4.

Each 64×64 image captures unique parasite positions and flagellar movements, producing biologically distinct samples rather than duplicates. Motion-based selection ensures temporal and spatial diversity, capturing multiple movement phases across videos.
**Algorithm 4** Training Sample Generation.**Require:** Enhanced frame $I_{enhanced}$, Parasite locations $P=\{p_{1},p_{2},\ldots ,p_{m}\}$
**Ensure:** Positive samples $P_{pos}$, Negative samples $P_{neg}$
 1: **for** each parasite location $p_{i}$ in *P* **do**
 2:    Extract crop with 50-pixel padding
 3:    Save as positive training sample
 4:**end for**
 5:**while** 
$|P_{neg}|<3$
**and** attempts <100 **do**
 6:    Generate random location (x,y)
 7:    **if** min_distance(x,y) to all parasites >80 px **then**
 8:        Extract 64×64 crop at (x,y)
 9:        Add to $P_{neg}$
 10:    **end if**
 11: **end while**
 12: **return** $P_{pos}$, $P_{neg}$


Training samples are extracted after frame enhancement, ensuring positive and negative samples reflect enhanced visibility. Table 5 summarizes the sampling strategy.

This implementation successfully processes 23 microscopic video sequences of *T. cruzi* parasites, generating balanced training datasets suitable for machine learning applications. The modular design allows for easy parameter tuning and extension to other parasitic organisms.

##### Dataset Splitting and Augmentation

After extracting positive and negative samples from all 23 videos, we prepared the dataset for model training and evaluation. The dataset was split into three parts using a 70-15-15 ratio: 70% for training (524 positive and 724 negative samples), 15% for validation (112 positive and 155 negative samples), and 15% for testing (113 positive and 156 negative samples).

To improve the model’s performance and reduce over-fitting, we applied data augmentation only to the training set. The validation and test sets were kept unchanged for fair evaluation. The augmentation techniques included horizontal and vertical flipping, rotation, brightness, and contrast adjustments, and adding Gaussian noise. These methods helped create a more diverse and balanced training dataset.

#### 3.2.3. Deep Learning Models and Training

We trained and evaluated three deep learning models for binary classification of parasites: MobileNetV2 [26], AlexNet [27], and VGG16 [28]. Each model was selected to represent a different family of convolutional neural networks (CNNs), balancing between efficiency, depth, and feature extraction capabilities. All models took RGB images resized to 224×224 as input, with pixel values normalized between 0 and 1. The training used a batch size of 32 and the Adam optimizer with a learning rate of 0.0001. We used binary cross-entropy as the loss function, and training was limited to 25 epochs. Early stopping was used with a patience of 7 epochs to avoid over-fitting if validation loss did not improve.

##### Model Selection

The selection of models for this study was motivated by the need to explore architectures with varying levels of complexity, computational efficiency, and representational capacity. This diversity enables a comprehensive evaluation of how different design philosophies in deep learning architectures influence the classification of parasites.

MobileNetV2 was chosen as a lightweight and efficient network architecture specifically designed for deployment in mobile and resource-limited environments. Its use of depthwise separable convolutions and inverted residuals allows it to achieve strong feature extraction with significantly reduced computational cost. With approximately only 3.4 million parameters, MobileNetV2 is particularly suitable for point-of-care diagnostic applications where fast inference and low power consumption are critical.

MobileNetV2 has also proven highly effective in related biomedical tasks. Prior studies report validation accuracies up to 99.9% [29] in malaria parasite detection, outperforming deeper models such as DenseNet, ResNet152V2, and NasNetLarge.

AlexNet, one of the earliest deep CNNs, was included to serve as a historical and structural benchmark. Although relatively shallow by modern standards, its convolutional layers pioneered the use of deep learning for image recognition and to provide insights into how classical architectures perform on this task. AlexNet contains around 61 million parameters, making it computationally heavier than MobileNetV2.

VGG16 was selected as a deeper architecture characterized by its uniform design of stacked convolutional layers. While computationally demanding, VGG16 is known for its strong feature extraction ability and robustness across many vision tasks. With approximately 138 million parameters, VGG16 provides a contrast to lightweight models, enabling an evaluation of how deeper, high-capacity networks compare in terms of their potential diagnostic utility.

This model selection strategy ensures that the study not only tests state-of-the-art efficient architectures but also examines the performance trade-offs introduced by classical and deeper CNN models. This balanced foundation allows for a comprehensive experimental evaluation in the subsequent sections. The quantitative differences between architectures (Table 6) enable systematic evaluation of efficiency trade-offs.

### 3.3. Video Processing for Object Detection

**Figure 5:**
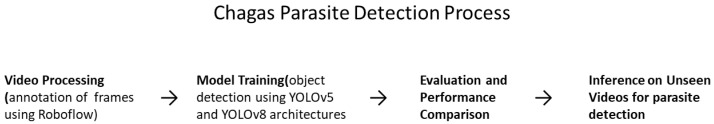
Chagas parasite detection process consisting of video annotation, model training with YOLOv5/YOLOv8, evaluation, and inference on unseen videos.

As illustrated in Figure 5, the proposed Chagas parasite detection pipeline involves four key stages: video processing, model training, evaluation, and inference.

**Figure 6:**
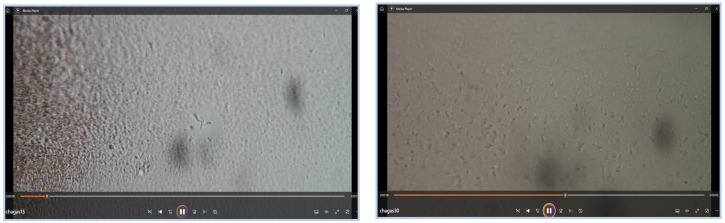
Examples of raw video data for parasite detection. This figure displays two representative frames from the dataset of microscopic videos. These frames illustrate the type of raw data—including background noise and varying lighting—that is used as input for the automated parasite detection system.

The dataset used in this study comprises a total of 48 microscopic video sequences, each containing biological samples potentially infected with *T. cruzi* parasites. These videos exhibit a wide range of conditions, including variations in background textures, lighting, image resolution, and parasite density. The diverse nature of the dataset ensures that the developed detection model can generalize across different real-world scenarios and environmental conditions observed in microscopic imaging. Figure 6 presents representative frames from the video dataset, illustrating the diversity of imaging conditions observed.

#### 3.3.1. Dataset Preparation

##### Manual Annotation and Ground Truth Generation

Out of the 48 available videos, a subset of 43 videos was selected for manual annotation. Each frame in these videos was carefully labelled by using Roboflow, an annotation tool that allows precise bounding box creation around visible *T. cruzi* parasites. The labelling process involved a thorough frame by frame inspection to ensure high annotation quality and consistency. The annotated data was exported in YOLO compatible format, enabling seamless integration into both YOLOv5 and YOLOv8 training pipelines.

##### Data Splitting Strategy

From 43 parasite detection videos, 895 annotated frames were extracted using the Roboflow platform. These frames were then divided into training and validation datasets using a simple 80:20 split. The training data was used to optimise the model parameters, while the validation data was employed to monitor performance during training and reduce the risk of over-fitting. This setup ensures that the model is evaluated on data it has not seen before, thereby enhancing its generalisation capability.

##### Model Training Using YOLO Architectures

In this phase of the study, the You Only Look Once (YOLO) framework was selected due to its demonstrated efficacy in comprehensive object detection. As a single-stage detection architecture, YOLO analyses the entire image in a single forward pass, allowing for the concurrent localisation and classification of multiple objects. This holistic processing significantly reduces information loss, which is particularly critical in the context of medical image interpretation.

To assess the effectiveness of the approach in parasite detection, two advanced iterations of the YOLO architecture were utilized: YOLOv5 and YOLOv8. YOLOv5 was adopted as a reliable baseline, while YOLOv8 incorporated state-of-the-art enhancements, including transformer-based components and decoupled detection heads to improve accuracy and generalisation. Both models were trained on a custom dataset, with essential hyper-parameters such as image resolution, batch size, and number of epochs carefully tuned to optimise performance. Each model variant was trained under identical hyper-parameter settings to ensure a fair comparison. Specifically, all models were trained for 140 epochs using a batch size of 8 and an input image size of 64×64 pixels.

##### Performance Evaluation

Model performance was assessed using standard object detection metrics, including precision, recall, and mean Average Precision (mAP) at multiple Intersection over Union (IoU) thresholds. Comparative evaluation between YOLOv5 and YOLOv8 was conducted using the validation set to determine the model with superior detection capabilities. Based on the evaluation results, the best performing model was selected for further inference on unannotated data.

##### Model Selection

We considered models that balance detection accuracy, computational efficiency, and suitability for resource-constrained deployment. YOLOv8 and YOLOv5 were selected as detection backbones because they represent complementary design philosophies within the same proven Ultralytics ecosystem, enabling a balanced evaluation of anchor-free and anchor-based approaches on the same dataset.

YOLOv8, the most recent generation (2023) of the YOLO family, introduces an anchor-free detection head and a refined C2f neck, which improve feature fusion and have been shown in prior benchmarks to achieve higher mAP scores than YOLOv5 for equivalent model sizes. For example, on the COCO dataset, YOLOv8-Nano reaches 37.3 mAP with only 3.2 M parameters, illustrating its combination of accuracy and computational efficiency [30]. YOLOv5-Nano, from an earlier generation (2020), remains an industry-standard backbone known for its exceptional inference speed, stability, and competitive accuracy. Its anchor-based design, built on a CSPDarknet53 backbone with a PANet neck, is highly effective for small-object detection. With a compact parameter count (2.6 M) and a mature, well-tested architecture, it is well-suited for real-time deployment on constrained edge hardware.

Including both models enables a rigorous architectural comparison: YOLOv8 represents a modern, anchor-free design optimized for performance and flexibility, while YOLOv5 offers a stable, speed-optimized architecture with proven practical value. This dual-model evaluation strengthens the generalizability of our findings across diverse deployment scenarios. Table 7 [30] shows Official Ultralytics benchmark results for the YOLOv8 and YOLOv5 models at 640 × 640 input resolution, showing mAP, inference speed, parameter count, and FLOPs. These values informed our choice of YOLOv8n and YOLOv5n for the experiments.

##### Model Architecture Variants

We evaluated six YOLO configurations: YOLOv5 (Nano, Small, Medium) and YOLOv8 (Nano, Small, Medium). The Nano versions prioritise speed and efficiency for lightweight deployment, the Small versions offer a balance between accuracy and performance, and the Medium versions provide higher detection accuracy with moderate computational requirements.

##### Inference on Unlabelled Videos

Following training and evaluation, the remaining 5 videos unannotated during the initial phase were used to assess the generalisation ability of the selected model. The trained YOLO model was applied to these unseen videos to perform real-time parasite detection. Each video was processed frame by frame, with bounding boxes drawn around detected parasite instances. The resulting annotated videos were saved and used for visual inspection, allowing researchers to analyse the spatial and temporal dynamics of parasite behaviour.

## 4. Experimental Results

This section presents the results of motion-based sample extraction and deep learning-based classification, followed by object detection using the YOLO framework. The performance is assessed through various metrics and comparisons.

### 4.1. Implementation Details

Experiments were conducted using Python 3.11.13, PyTorch version 2.6.0+cu124, and TensorFlow version 2.18.0 in the Google Colab integrated programming environment. Both training and testing were carried out on an NVIDIA Tesla T4 (15,360 MiB) GPU for efficient computations. This setup enabled high-performance deep learning experiments, leveraging the GPU hardware accelerator to significantly reduce training time and support resource intensive tasks under a Windows 11 host environment.

### 4.2. Evaluation Metrics

In this study, the performance of the models was assessed using distinct sets of evaluation metrics. These metrics provide a comprehensive view of each model’s effectiveness across different tasks, ensuring robust performance in real-world applications. The classification performance was evaluated using accuracy, Equation (1), precision, Equation (2), recall, Equation (3), and F1-score, Equation (4). In addition, the object detection capability was quantified using the mean Average Precision (mAP) computed as in Equation (5).

(1) $$\begin{array}{cc} \mathrm{Accuracy} & =\frac{\mathrm{TP}+\mathrm{TN}}{\mathrm{TP}+\mathrm{FP}+\mathrm{FN}+\mathrm{TN}}, \end{array}$$

(2) $$\begin{array}{cc} \mathrm{Precision} & =\frac{\mathrm{TP}}{\mathrm{TP}+\mathrm{FP}}, \end{array}$$

(3) $$\begin{array}{cc} \mathrm{Recall} & =\frac{\mathrm{TP}}{\mathrm{TP}+\mathrm{FN}}, \end{array}$$

(4) $$\begin{array}{cc} F1\;\mathrm{score} & =\frac{2\times \mathrm{Precision}\times \mathrm{Recall}}{\mathrm{Precision}+\mathrm{Recall}}, \end{array}$$

(5) $$\begin{array}{cc} \mathrm{mAP} & =\frac{1}{N}\sum_{i=1}^{N}{\mathrm{AP}}_{i}, \end{array}$$

where TP (True Positives) represents the correctly predicted positive samples, TN (True Negatives) represents the correctly predicted negative samples, FP (False Positives) incorrectly predicted positives, and FN (False Negatives) incorrectly predicted negatives.

### 4.3. Motion-Based Parasite Detection and Sample Classification

**Figure 7:**
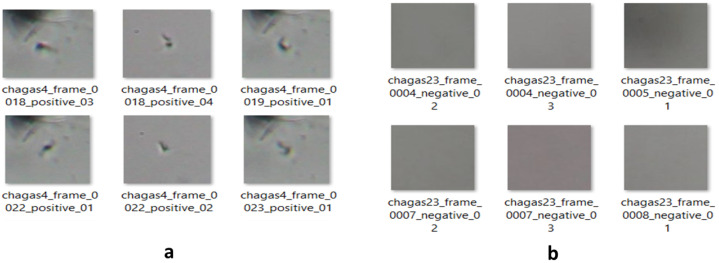
Representative sample images extracted from microscopic video sequences. The figure shows positive samples containing visible *T. cruzi* parasites (**a**), and negative samples without parasites (**b**). These images were used for training and evaluating the classification models.

The proposed motion-based framework was applied to 23 microscopic video sequences containing *T. cruzi* parasites. Using adaptive frame differencing and morphological filtering, a total of 1784 samples were extracted, comprising 749 positive (with parasites) and 1035 negative (without parasites) samples. Representative examples of these extracted samples are illustrated in Figure 7, which shows both positive and negative cases used for training and evaluating the classification models.

#### Deep Learning Classification Results

Three deep learning architectures were trained and evaluated for binary classification of microscopic images of *T. cruzi* parasites: MobileNetV2, AlexNet, and VGG16.

**Figure 8:**
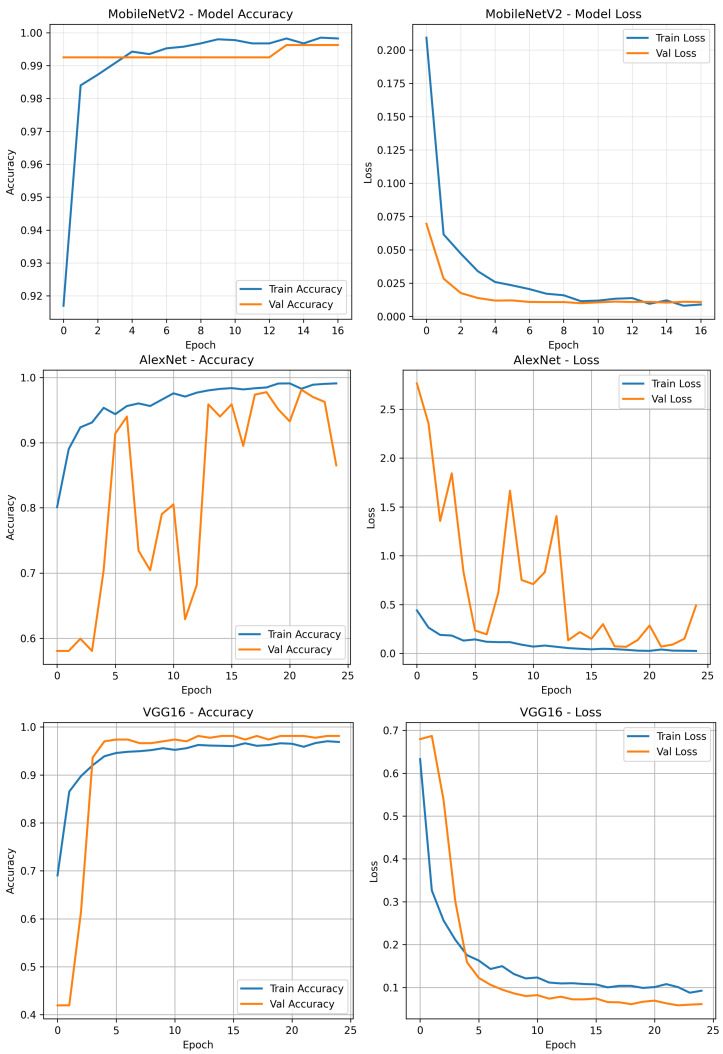
Training results of MobileNetV2, AlexNet, and VGG16.

To analyse model performance, Figure 8 illustrates the training results of MobileNetV2, AlexNet, and VGG16. It shows the changes in training and validation accuracy and loss across epochs, which help evaluate each model’s convergence behaviour and generalisation performance.

Table 8 summarises the training and validation performance of the evaluated deep learning models. Among the three architectures, MobileNetV2 demonstrated the most effective convergence behaviour, attaining a final training accuracy of 99.86% and a validation accuracy of 99.25%. The minimal discrepancy between training and validation loss values (0.0113 and 0.0100, respectively) indicates excellent generalisation capability with negligible over-fitting.

AlexNet also achieved strong results, with a training accuracy of 98.42% and a validation accuracy of 97.75%. Its validation loss of 0.0686 remained within an acceptable range, supporting the model’s ability to generalise effectively.

VGG16 reached a training accuracy of 96.41%, and like AlexNet, recorded a validation accuracy of 97.75%. However, its relatively higher training loss of 0.1039 may suggest slower convergence or the need for additional regularisation or training epochs. Despite this, the model still exhibited good generalisation to unseen data.

Overall, these results highlight the robustness and efficiency of MobileNetV2, making it a strong candidate for deployment in real-time or resource-constrained settings, particularly in biomedical image classification tasks. Table 9 presents the detailed evaluation metrics obtained on the test set for the three deep learning models: MobileNetV2, AlexNet, and VGG16. The performance was measured using accuracy, precision, recall, F1-score, and loss. These results provide insight into the generalisation ability and robustness of each model in classifying *T. cruzi* parasite images.

**Figure 9:**
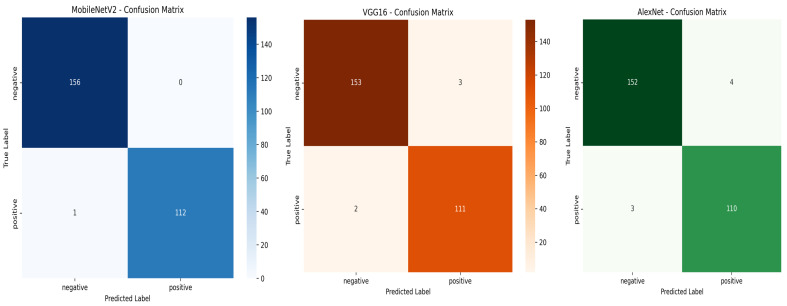
Confusion matrices for MobileNetV2, VGG16, and AlexNet on the test set.

Among the evaluated models, MobileNetV2 achieved the highest overall performance with an accuracy of 99.63%, perfect precision (100%), and an AUC-ROC score of 1.0000, indicating outstanding classification ability. VGG16 and AlexNet also achieved good and acceptable performance across all metrics. These results, illustrated in the confusion matrices shown in Figure 9, highlight the effectiveness of deep learning methods in classifying *T. cruzi* parasites from microscopic images.

MobileNetV2 showed excellent classification capability, correctly identifying all negative samples (TN = 156, FP = 0) and misclassifying only one positive sample (FN = 1). This resulted in the highest recall (99.12%) and perfect precision (100%).

VGG16 also performed well with only three total errors (three FP and two FN), maintaining strong overall performance and a good balance between precision and recall.

AlexNet misclassified seven samples (four FP and three FN), which slightly reduced its precision and recall, but it still achieved reliable classification with minimal over-fitting.

**Figure 10:**
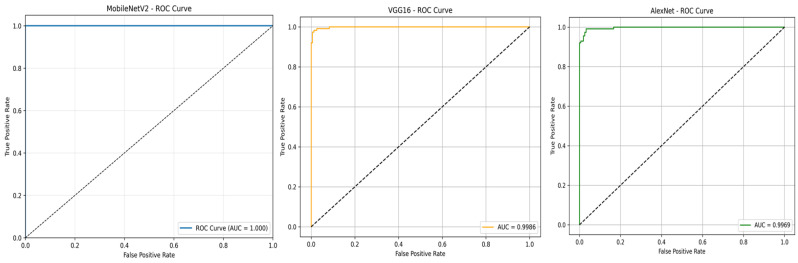
ROC curves for MobileNetV2, VGG16, and AlexNet.

To further evaluate the classification performance of the three models, the Receiver Operating Characteristic (ROC) curves were plotted, as shown in Figure 10. MobileNetV2 achieved an AUC-ROC of 1.00, confirming its outstanding capability in distinguishing positive and negative T. cruzi parasite images. VGG16 and AlexNet obtained AUC-ROC scores of 0.9986 and 0.9969, respectively, reflecting strong classification performance with minimal misclassification.

To assess the models’ suitability for real-time applications, inference speed was measured on the test dataset. MobileNetV2 achieved an average inference time of 13.6 ms per image, AlexNet 3.6 ms per image, and VGG16 7.8 ms per image. These results indicate that all models are suitable for practical deployment, with MobileNetV2 providing an optimal balance between high classification accuracy and fast prediction speed (Table 10).

### 4.4. Video-Based Parasite Localization Using YOLO Detection Models

To evaluate the effectiveness of object detection models in localizing T.cruzi parasites within microscopic videos, a total of 43 annotated videos were used for training and validation. Each video was processed frame by frame, and parasite instances were manually labelled using bounding boxes. The annotated data was used to train multiple YOLO-based models, namely YOLOv5 and YOLOv8, in three configurations each (Nano, Small, and Medium). The validation set comprised a diverse range of frames with varying parasite densities, lighting conditions, and background textures, closely simulating real-world microscopy environments.

#### 4.4.1. Training Performance for Video-Based Parasite Detection

The performance of the trained models was evaluated using widely accepted object detection metrics, including precision (P), recall (R), mean Average Precision at IoU threshold 0.5 (mAP@0.5), and mean Average Precision averaged over IoU thresholds from 0.5 to 0.95 (mAP@0.5:0.95). These metrics provide a comprehensive view of the model’s localisation accuracy and its robustness across different levels of detection strictness. Table 11 summarises the detection performance of all YOLO variants on the validation dataset.

As shown in Table 11, the YOLOv8-Nano model achieved the highest mAP@0.5:0.95 score (0.226), indicating a strong balance between localisation precision and robustness across various IoU thresholds. Interestingly, YOLOv5-Nano achieved the highest mAP@0.5 (0.604), suggesting it performed slightly better at standard IoU evaluation compared to its counterparts.

YOLOv5-Nano and YOLOv8-Nano also showed competitive results in terms of precision and recall, making them suitable candidates for deployment in resource-constrained environments due to their efficiency and lightweight architecture.

**Figure 11:**
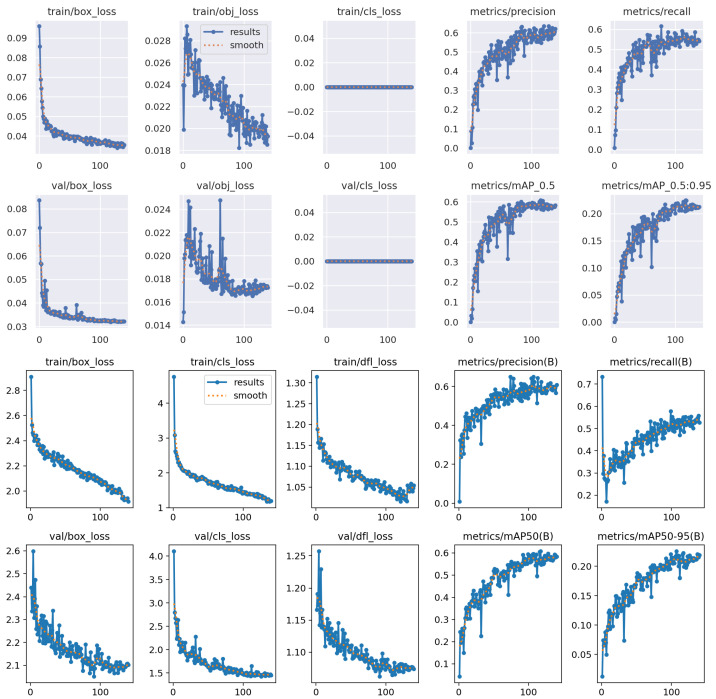
Comparison of training and validation performance for YOLOv5-Nano and YOLOv8-Nano models.

It is also important to note that, in general, the detection results were modest and did not reach exceptionally high scores. This is likely due to the inherent challenges of the dataset: many video frames contained unclear or noisy backgrounds, poor lighting conditions, and parasites that were barely visible. These factors negatively impacted the model’s ability to consistently detect and localize *T. cruzi* parasites, especially in more challenging frames.This observation is supported by the comparative training and validation performance curves shown in Figure 11, which illustrate fluctuations in learning stability and detection accuracy across both YOLOv5-Nano and YOLOv8-Nano models.

The training curves of the YOLOv5-Nano model demonstrate steady and consistent improvement across all key metrics. Specifically, the loss components—including the bounding box regression loss and the objectness loss (which measures the confidence of object presence within predicted regions)—show a gradual and stable decrease, indicating effective learning and convergence. Both precision and recall steadily increase throughout the training process, while the mAP@0.5 and mAP@0.5:0.95 metrics approach competitive values. Overall, the training appears smooth and well regularised, with no signs of over-fitting or instability. The results suggest that YOLOv5-Nano is highly efficient and well suited for this task, especially considering its lightweight architecture.

Similarly, the YOLOv8-Nano model exhibits strong convergence behaviour. All three loss components, box loss, classification loss, and distribution focal loss, decrease progressively during training, reflecting proper optimisation. Precision and recall also improve consistently, and both mAP metrics show a steady upward trend. However, the initial loss values are relatively high compared to YOLOv5-Nano, possibly due to architectural complexity or dataset sensitivity. Although the final performance is stable and competitive, YOLOv8-Nano did not surpass YOLOv5-Nano in key metrics.

While both models demonstrate effective learning, YOLOv5-Nano slightly outperforms YOLOv8-Nano in terms of detection accuracy and training stability. This makes YOLOv5-Nano a more favourable option for resource-constrained environments and real-time microscopic parasite detection.

#### 4.4.2. Qualitative Testing on Unseen and External Videos

**Figure 12:**
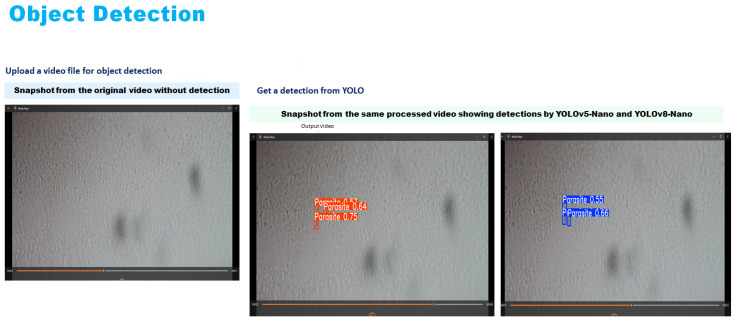
Processed video output—YOLOv5 and YOLOv8 detections.

To further assess the real-world applicability of the proposed detection system, qualitative inference was conducted on two additional video sources. The evaluation involved five unseen videos from the original dataset that were excluded from the training and validation phases, as well as one external outdoor video captured independently under high-quality imaging conditions. Visual inspection of the detection outputs was used to assess the model’s behaviour and effectiveness across different environmental conditions and video qualities. As shown in Figure 12, the results correspond to unseen videos from the original dataset.

The best-performing models, YOLOv5-Nano and YOLOv8-Nano, were selected for this qualitative assessment based on their superior performance during the quantitative evaluation phase. Both models underwent inference testing on the unseen video datasets to evaluate their generalisation capabilities and real-world applicability. Both models successfully detected and localised multiple parasite instances, demonstrating robust performance even under challenging conditions such as motion blur, variable lighting, and cluttered or noisy backgrounds. The detection results showed strong consistency across different frames and time intervals, indicating good temporal stability of the detection system.

**Figure 13:**
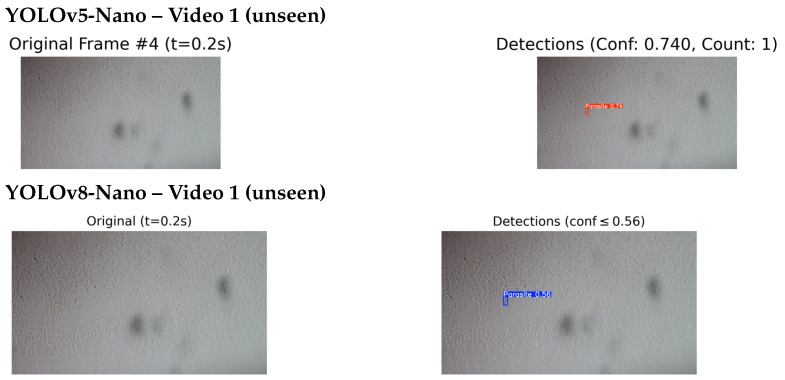
Comparison of detection results on unseen Video 1: YOLOv5-Nano (top) and YOLOv8-Nano (bottom) applied to the same video sequence. This comparison evaluates the detection performance of each model under identical conditions.

**Figure 14:**
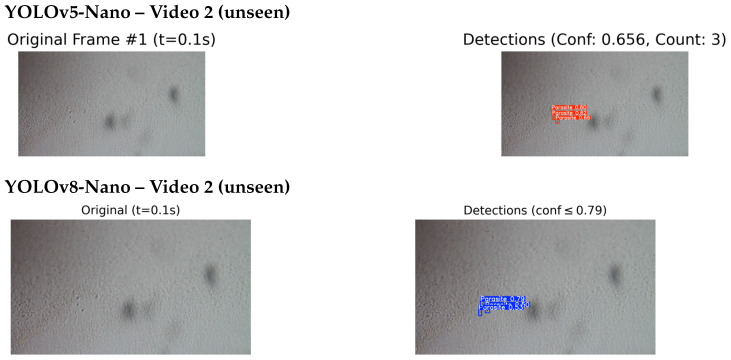
Comparison of detection results on unseen Video 2: YOLOv5-Nano (top) and YOLOv8-Nano (bottom) applied to the same video sequence. This comparison evaluates the detection performance of each model under identical conditions.

Figure 13 and Figure 14 present a side by side comparison of the YOLOv5-Nano and YOLOv8-Nano models applied to unseen microscopic videos containing *T. cruzi* parasites.

**Figure 15:**
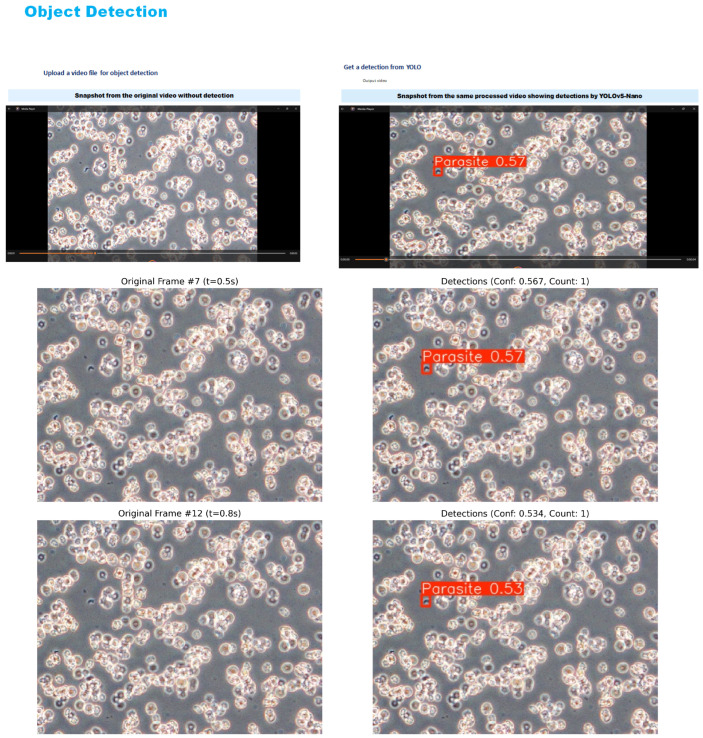
Processed video output – YOLOv5 detections for videos taken from Martin et al.’s [31] work.

In addition, Figure 15 presents the output for an external video captured under different lighting and background conditions.

In Video 1, the YOLOv5-Nano model achieved a higher confidence score of 0.74, compared to YOLOv8-Nano’s 0.56, indicating stronger detection certainty on the same frame and timestamp. However, in Video 2, YOLOv8-Nano produced more accurate bounding boxes and achieved a higher confidence score of 0.79, while YOLOv5-Nano reached 0.656.

In both test cases, the models were evaluated on video frames with similar visual challenges, such as low contrast and background noise. This comparison highlights how each model architecture performs under identical or near identical conditions, providing insights into their generalisation capabilities on unseen microscopic sequences. While a few missed detections were observed in frames with low contrast or poor visibility reflecting known challenges in the dataset, these instances were relatively infrequent and did not substantially affect overall detection performance. The system demonstrated strong qualitative results, with most detections appearing visually accurate and consistent with expert expectations.

In this study, we worked with newly collected data for training and evaluation, without direct comparison to previous works. However, to assess generalization, we utilized a microscopic video from the publicly available dataset by Martin et al. [31]. This video was recorded under ideal imaging conditions using an Olympus CKX41 microscope at 400× or 200× magnification. It clearly shows a motile trypomastigote actively swimming among red blood cells, making it an optimal reference for evaluating our model’s performance under controlled conditions.

In this high-quality setting, the detection system demonstrated good and accurate performance, producing tightly aligned bounding boxes around parasite-like structures.

Detection confidence was consistently high, with average confidence scores exceeding 0.5 in most cases. This reflects the model’s ability to generalise effectively and maintain robust performance when applied to structured, real-world video data. The results from the outdoor test demonstrate that the detection system performs exceptionally well under ideal conditions, indicating strong potential for deployment in controlled or high-quality recording environments.

## 5. Discussion and Conclusions

This study introduces a novel dual-stage framework for automated *T. cruzi* detection that strategically combines motion-based preprocessing with deep learning to address critical limitations in Chagas disease diagnostics. The innovative motion analysis pipeline, utilizing frame differencing, morphological filtering, and DBSCAN clustering across 23 microscopic videos, successfully exploits parasite motility as a discriminative feature, proving particularly effective in challenging conditions with low contrast, uneven illumination, and complex backgrounds where traditional appearance-based methods fail. The motion-guided sample extraction approach enabled MobileNetV2 to achieve exceptional performance metrics (99.63% accuracy, 100% precision, 99.12% recall, and AUC-ROC of 1.0), validating the robustness of the preprocessing strategy.

The novelty of our approach lies not in individual techniques, but in their strategic integration for parasitological applications. Unlike existing methods that treat motion as auxiliary information, our framework positions parasite motility as the primary detection signal, fundamentally changing the diagnostic approach from appearance-based to behaviour-based identification. This motion-first strategy enables reliable detection even when morphological features are compromised by poor imaging conditions, addressing a critical gap in current automated diagnostic tools for Chagas disease. Subsequently, a comprehensive evaluation of six YOLO configurations, YOLOv5 and YOLOv8 across Nano, Small, and Medium variants, identified YOLOv8-Nano and YOLOv5-Nano as optimal candidates, offering a strong balance between detection accuracy and computational efficiency. Both models demonstrated robust generalization when applied to previously unseen microscopic videos, including challenging outdoor scenarios, where YOLOv8-Small achieved a peak detection confidence of 0.79 with precise localization.

The clinical significance of this framework lies in its ability to deliver an objective, reproducible, and lightweight solution for automated Chagas disease diagnosis. This is particularly valuable in resource-limited environments, where traditional manual microscopy is hindered by low sensitivity and subjective interpretation. By supporting real-time video processing with high detection accuracy (99.63% vs. 72–96.4% for existing static methods), the system is well suited for mobile health applications and point of care diagnostics. Its lightweight design enables rapid processing, with motion detection completing in 3.27 s per video (75.3 s for 23 videos) and classification inference, using the best-performing model MobileNetV2, requiring only 13.6 milliseconds per image (434 ms per 32-image batch), making it suitable for real-time deployment.

Future research should aim to expand the dataset to encompass a broader range of microscope types, magnification levels, and imaging conditions. Incorporating temporal consistency mechanisms may help further reduce false positives. Overall, this work represents a significant advancement in automated parasitological diagnostics, bridging classical motion-based computer vision with modern deep learning to deliver a practical, scalable, and impactful tool. It brings reliable parasite screening closer to routine clinical implementation and has the potential to improve healthcare outcomes in regions where Chagas disease remains a pressing public health concern.

## Figures and Tables

**Table 1:** Frame differencing parameters.

Parameter	Value	Description
Motion Threshold (*T*)	25	∼10% of 8-bit intensity range
Gaussian Kernel	5×5	Noise reduction filter

**Table 2:** Enhancement parameters.

Technique	Parameters	Purpose
CLAHE	clip_limit = 2.0, tile_size = 8×8	Contrast enhancement
Colour Space	LAB (L-channel only)	Preserve chromaticity

**Table 3:** Detection and clustering parameters.

Component	Parameter	Value	Purpose
Contour Analysis	Min Area	50 pixels	Filter Noise
Morphological	Kernel Size	2×2 ellipse	Shape Refinement
DBSCAN	Epsilon (ε)	30 pixels	Clustering Radius
DBSCAN	Min Samples	2 points	Min Cluster Size
Sample Generation	Negative Distance	80 pixels	Avoid Parasite Regions

**Table 4:** Time analysis of motion preprocessing stage.

Total Time	Videos	Avg. Time/Video
75.3 s	23	3.27 s

**Table 5:** Sample generation strategy.

Sample Type	Count per Frame	Size	Constraints
Positive	Variable	64×64 + 50 px padding	Around detected parasites
Negative	3	64×64	>80 px from any parasite

**Table 6:** Comparison of model complexity: parameters and FLOPs.

Model	Parameters (M)	FLOPs (G)
MobileNetV2	3.4	0.30
AlexNet	61.0	0.72
VGG16	138.0	15.3

**Table 7:** Benchmark results for YOLOv8 and YOLOv5 models.

Model	Size (Pixels)	mAP^val^ 50–95	Speed CPU ONNX (ms)	Speed T4 TensorRT (ms)	Params (M)	FLOPs (B)
YOLOv8n	640	37.3	80.4	1.47	3.2	8.7
YOLOv8s	640	44.9	128.4	2.66	11.2	28.6
YOLOv8m	640	50.2	234.7	5.86	25.9	78.9
YOLOv8l	640	52.9	375.2	9.06	43.7	165.2
YOLOv8x	640	53.9	479.1	14.37	68.2	257.8
YOLOv5n	640	28.0	73.6	1.12	2.6	7.7
YOLOv5s	640	37.4	120.7	1.92	9.1	24.0
YOLOv5m	640	45.4	233.9	4.03	25.1	64.2
YOLOv5l	640	49.0	408.4	6.61	53.2	135.0
YOLOv5x	640	50.7	763.2	11.89	97.2	246.4

**Table 8:** Training and validation performance of deep learning models.

Model	Train Accuracy (%)	Train Loss	Val Accuracy (%)	Val Loss
MobileNetV2	99.86	0.0113	99.25	0.0100
AlexNet	98.42	0.0412	97.75	0.0686
VGG16	96.41	0.1039	97.75	0.0581

**Table 9:** Test performance comparison of deep learning models.

Model	Accuracy (%)	Precision (%)	Recall (%)	F1-Score (%)	Loss	AUC
MobileNetV2	99.63	100.00	99.12	99.56	0.0099	1.00
AlexNet	97.40	96.49	97.35	96.92	0.0794	0.9969
VGG16	98.14	97.37	98.23	97.80	0.0730	0.9986

**Table 10:** Inference times of the evaluated deep learning models.

Model	Inference Time (ms/Image)
MobileNetV2	13.6
AlexNet	3.6
VGG16	7.8

**Table 11:** Detection performance comparison of YOLOv5 and YOLOv8 models on the validation dataset.

Model	Precision (P)	Recall (R)	mAP@0.5	mAP@0.5:0.95
YOLOv5-Nano	0.581	0.575	0.604	0.224
YOLOv5-Small	0.631	0.525	0.590	0.215
YOLOv5-Medium	0.549	0.559	0.595	0.219
YOLOv8-Nano	0.651	0.504	0.595	0.226
YOLOv8-Small	0.573	0.544	0.592	0.214
YOLOv8-Medium	0.560	0.515	0.553	0.208

## Data Availability

The raw data supporting the conclusions of this article will be made available by the authors on request.

## Abbreviations

The following abbreviations are used in this manuscript:

AUC	Area under ROC Curve
CIR	Laboratory of Zoonotic Diseases
CNN	Convolutional Neural Network
DL	Deep Learning
FP	False Positive
FN	False Negative
IACUC	The Institutional Animal Care and Use Committee of the University
IoU	Intersection over Union
JImaging	Journal of Imaging
mAP	Mean Average Precision
ROC	Receiver Operating Characteristic
ROI	Region of Interest
TP	True Positive
*T. cruzi*	*T. cruzi*
TN	True Negative
